# p38γ overexpression promotes osteosarcoma cell progression

**DOI:** 10.18632/aging.103708

**Published:** 2020-09-24

**Authors:** Ce Shi, Wei-Nan Cheng, Yin Wang, Da-Zhuang Li, Li-Na Zhou, Yu-Cheng Zhu, Xiao-Zhong Zhou

**Affiliations:** 1 Department of Orthopedics, The Second Affiliated Hospital of Soochow University, Suzhou, China; 2 Department of Orthopedics, The Affiliated Suqian Hospital of Xuzhou Medical University, Suqian, China; 3 Department of Orthopedics, The First Affiliated Hospital of Xiamen University, Xiamen, China; 4 Jiangsu Key Laboratory of Neuropsychiatric Diseases and Institute of Neuroscience, Soochow University, Suzhou, China; 5 Department of Radiotherapy and Oncology, Affiliated Kunshan Hospital of Jiangsu University, Kunshan, China

**Keywords:** osteosarcoma, p38γ, molecularly targeted therapy

## Abstract

Osteosarcoma (OS) is the most common primary bone malignancy in the adolescent population. Recent studies demonstrate that p38 gamma (p38γ) phosphorylates retinoblastoma (Rb) to promote cyclin expression, cell-cycle entry and tumorigenesis. Studying the potential function of p38γ in human OS, we show that *p38*γ mRNA and protein expression are significantly elevated in OS tissues and OS cells, whereas its expression is relatively low in normal bone tissue and in human osteoblasts/osteoblastic cells. Knockdown of p38γ in established (U2OS) and primary human OS cells potently inhibited cell growth, proliferation, migration and invasion, while promoting cell apoptosis. Furthermore, CRISPR/Cas9-induced p38γ knockout inhibited human OS cell progression *in vitro*. Conversely, ectopic overexpression of p38γ in primary human OS cells augmented cell growth, proliferation and migration. Signaling studies show that retinoblastoma (Rb) phosphorylation and cyclin E1/cyclin A expression were decreased following p38γ shRNA knockdown and knockout, but increased after ectopic p38γ overexpression. Collectively, these results show that p38γ overexpression promotes human OS cell progression.

## INTRODUCTION

Osteosarcoma (OS) is the most common primary and malignant bone tumor detected in children, adolescents, and young adults [1]. The current treatment options, including systemic chemotherapy and local control surgery, have significantly improved OS survival to 70% from the 1970s [1]. However, using these approaches, studies have shown that survival has reached a plateau with little to no further improvement of overall survival [1, 2], and the prognosis of patients with high-grade, metastatic and recurrent human OS remains at 20 to 30% survival [1].

To improve the outcome for OS patients there is an urgent need to understand the pathological mechanisms of OS progression [3–5]. Recent advances in the molecular genetics of OS have provided new therapeutic approaches for its treatment [2, 6–8]. Tomás-Loba et al., reported that p38γ is a novel cyclin-dependent kinase (CDK)-like kinase that facilitates cell-cycle entry and liver tumorigenesis [9]. p38γ phosphorylates the tumor suppressor protein retinoblastoma (Rb), causing increased expression of cyclin E1 and cyclin A, thereby promoting tumor cell proliferation and progression [9]. Contrarily, p38γ silencing or knockout (KO) leads to suppression of tumor cell progression [9]. Chen et al., reported that overexpression of p38γ in human renal cell carcinoma (RCC) tissues is required for tumor cell growth, proliferation and migration [10]. Furthermore, Su et al., demonstrated that targeting p38γ in colorectal cancer resulted in decreased cancer growth and apoptosis [11]. The results of this study show that p38γ overexpression promotes human OS cell progression, and that p38γ is a promising therapeutic target for treating human OS.

## RESULTS

### p38γ expression is elevated in human OS tissues and OS cells

**Figure 1:**
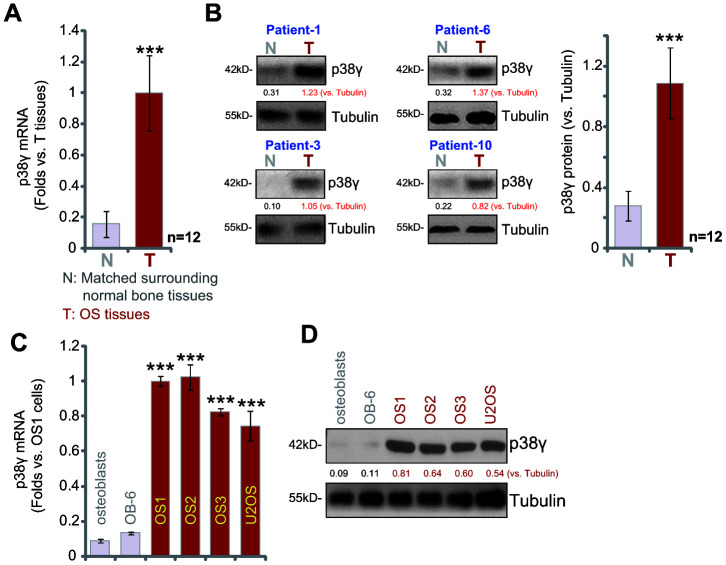
**p38γ expression is elevated in human OS tissues and OS cells.** Expression of *p38γ* mRNA (**A**, **C**) and protein (**B**, **D**) in twelve (n=12, derived from twelve different OS patients) primary human osteosarcoma tissues (“T”) and matched surrounding normal bone tissues (“N”), as well as in U2OS established OS cells, primary human OS cells (OS1/OS2/OS3, derived from three different OS patients), OB-6 osteoblastic cells, and primary human osteoblasts are shown. Expression of listed proteins was quantified and normalized to the loading control (**B**, **D**). Data presented as mean ± standard deviation (SD). *** *p*< 0.001 vs. “N” tissues/osteoblasts.

Examining p38γ expression, a total of twelve (n=12) different OS tissues derived from primary human OS patients were tested. qPCR analysis of mRNA expression revealed that p38γ mRNA levels in OS tumor tissues (“T”) were increased over six fold compared to matched surrounding normal bone tissue (“N”) (P<0.001, Figure 1A). Testing p38γ protein expression by Western blotting analysis confirmed a significant upregulation of p38γ protein in OS tumor tissues (Figure 1B, P<0.001 vs. “N” tissues). Similarly, p38γ mRNA (Figure 1C) and protein expression (Figure 1D) are also significantly higher in the U2OS OS cell line and primary human OS cells. The human OS cells were derived from three primary human OS patients, namely OS1, OS2 and OS3 (see Methods, Figure 1C, 1D). In contrast, p38γ mRNA and protein expression were relatively low in OB-6 human osteoblastic cells and primary human osteoblasts (Figure 1C, 1D). These results show that p38γ expression is elevated in human OS tissues and OS cells.

### p38γ shRNA inhibits human OS cell viability, growth, proliferation, migration and invasion

**Figure 2:**
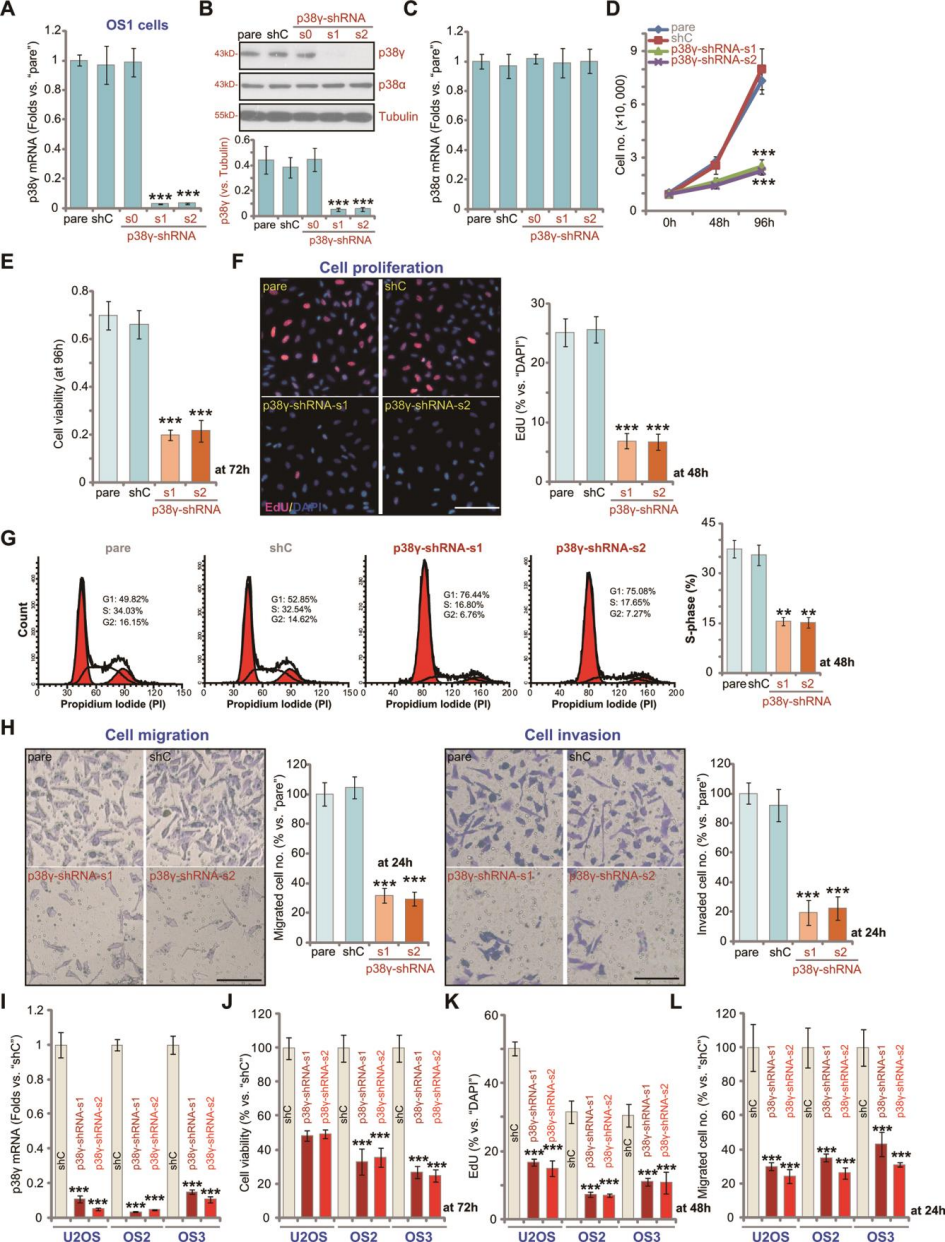
**p38γ shRNA inhibits human OS cell viability, growth, proliferation, migration and invasion.** Human OS cells, including OS1/OS2/OS3 primary OS cells (derived from three different OS patients) and the established U2OS cells, with scramble control shRNA (“shC”) or the applied p38γ shRNA (p38γ-shRNA-s0/s1/s2), were cultured and the expression of listed genes tested by qPCR and Western blotting assays (**A**–**C**, **I**); Cell growth (cell counting assay, **D**), viability (measuring CCK-8 viability OD, **E**, **J**) and proliferation (measuring EdU ratio, **F**, **K**) as well as cell cycle distribution (**G**), cell migration (“Transwell” assay, **H**, **L**) and invasion (“Matrigel Transwell” assay, **H**) were tested after incubation for applied time periods. “pare” indicated parental control cells (same for all Figures). For EdU staining assays, five random views with total 500 cell nuclei from each treatment were included to calculate the EdU/DAPI ratio (same for all Figures). For “Transwell”/“Martial Transwell” assays, in each condition five random views were included to calculate the average number of migrated/invaded cells (same for all Figures). For all the functional assays the same number of viable cells from the different genetic treatments were seeded initially onto each well or each dish (at 0h, same for all Figures). Expression of listed proteins was quantified and normalized to the loading control (**B**). Data presented as mean ± standard deviation (SD, n=5). ** *p*< 0.01 vs. “shC” cells. ** *p*< 0.001 vs. “shC” cells. Experiments in this figure were repeated five times. Bar=100 μm (**F**, **H**).

To investigate the impact of p38γ in OS cell function we generated three stable cell lines with shRNAs targeting p38γ. A set of three lentiviral p38γ shRNAs, with non-overlapping sequences, p38γ-shRNA-s0/s1/s2, were individually transduced into the primary human cells derived patient OS1. As shown, p38γ-shRNA-s1 and p38γ-shRNA-s2 reduced *p38γ* mRNA expression by greater than 95% (Figure 2A, *P*<0.001 vs. control shRNA/shC), resulting in knockdown of p38γ protein in p38γ-shRNA-s1/s2-expressing OS1 cells (Figure 2B, *P*<0.001 vs. shC). In contrast, p38γ-shRNA-s0 did not alter *p38γ* mRNA and protein expression in OS1 cells (Figure 2A, 2B, *P*>0.05 vs. shC). The p38γ shRNAs failed to change expression of *p38α* mRNA (Figure 2C, *P*>0.05 vs. shC) and protein (Figure 2B, *P*>0.05 vs. shC) in OS1 cells.

Cell growth curve results, Figure 2D, demonstrated that the growth of p38γ-shRNA-s1/s2 OS1 cells was significantly slower than shC control cells (*P*<0.001). Examining cell viability, using a CCK-8 assay which reflects proliferative ability, p38γ silencing resulted in a 60-70% reduction in viability (Figure 2E, *P*<0.001 vs. shC). In agreement, nuclear EdU incorporation (% vs. DAPI) was sharply decreased in p38γ-silenced OS1 cells (Figure 2F, *P*<0.001 vs. shC). Corroborating these results, FACS analysis demonstrated that p38γ silencing disrupted cell cycle progression, causing G1-S arrest (Figure 2G).

Further functional studies showed that silencing of p38γ potently inhibited OS1 cell *in vitro* migration and invasion, tested using Transwell (Figure 2H, the left panel) and Martial Transwell assays (Figure 2H, the right panel), respectively. Similar results were observed for the primary human OS cells-derived from patients, OS2 and OS3, as well as in the established U2OS cell line. In these cells each of the applied p38γ shRNAs (p38γ-shRNA-s1 and p38γ-shRNA-s2) robustly inhibited cell viability (CCK-8 OD at 72h, Figure 2J, *P*<0.001 vs. shC), proliferation (nuclear EdU ratio at 48h, Figure 2K, *P*<0.001 vs. shC) and migration (at 24h, Figure 2L, *P*<0.001 vs. shC). Collectively, these results show that in human OS cells p38γ silencing inhibited cell viability, growth, proliferation, migration and invasion.

### p38γ shRNA provokes apoptosis in human OS cells

**Figure 3:**
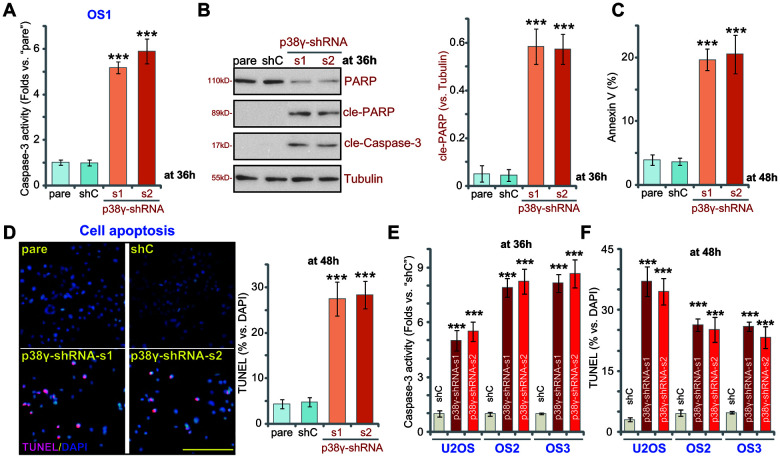
**p38γ shRNA provokes apoptosis in human OS cells.** Human OS cells, including OS1/OS2/OS3 primary cells and the established U2OS cells, with scramble control shRNA (“shC”) or the applied p38γ shRNA (p38γ-shRNA-s1/s2), were cultured for applied time periods, the relative caspase-3 activity was tested (**A**, **E**), with expression of apoptosis-associated proteins examined by Western blotting (**B**); Cell apoptosis was tested by Annexin V FACS (**C**) and nuclear TUNEL staining (**D**, **F**) assays, and results were quantified. Expression of listed proteins was quantified and normalized to the loading control (**B**). Data presented as mean ± standard deviation (SD, n=5). *** *p*< 0.001 vs. “shC” cells. Experiments in this figure were repeated five times. Bar=100 μm (**D**).

As inhibition of proliferation often results in apoptosis, we therefore examined apoptosis activation in p38γ-depleted OS cells. In p38γ-shRNA-s1/s2OS1 cells (see Figure 2) caspase-3 activity was significantly elevated (Figure 3A, *P*<0.001 vs. shC). Furthermore, increased levels of cleaved-caspase-3 and cleaved-PARP were also detected in p38γ-silenced OS1 cells (Figure 3B, *P*<0.001 vs. shC). Confirming apoptosis, p38γ knockdown significantly increased the ratio of Annexin V-positive OS1 cells (Figure 3C; *P*<0.001 vs. shC) and potently increased the ratio of TUNEL-positive nuclei (% vs. DAPI, Figure 3D, *P*<0.001 vs. shC). These results demonstrate that p38γ knockdown induces significant apoptosis activation in OS1 cells. The identical results were observed in primary OS2/ OS3 cells and in established U2OS cells, where shRNA-induced silencing of p38γ (by p38γ-shRNA-s1/p38γ-shRNA-s2, see Figure 2) increased caspase-3 activity (Figure 3E, *P*<0.001 vs. shC) and the ratio of TUNEL-positive nuclei (Figure 3F, *P*<0.001 vs. shC).

### p38γ KO inhibits human OS cell progression *in vitro*

**Figure 4:**
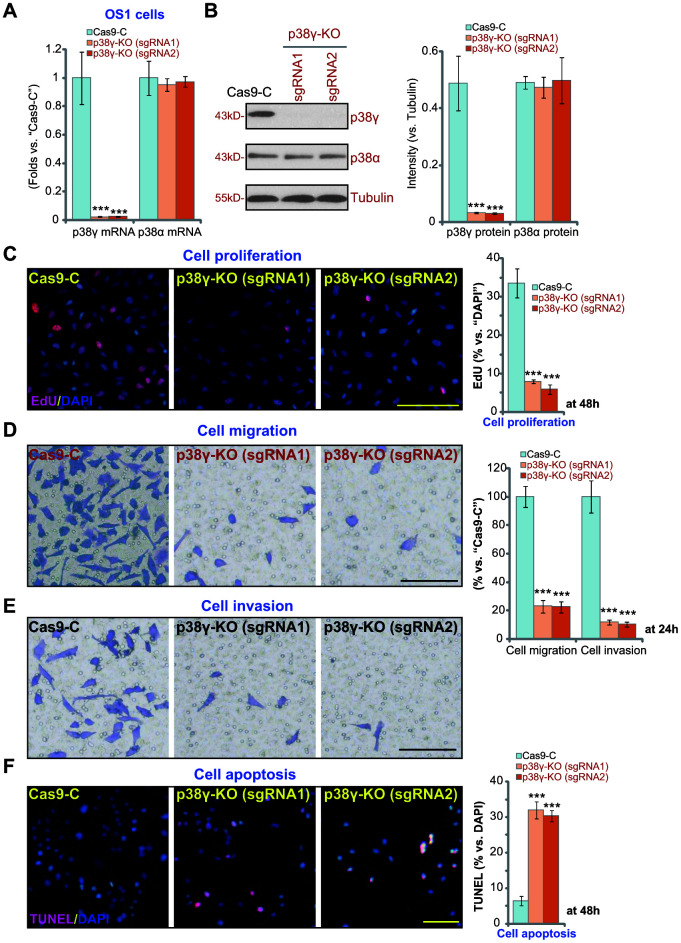
**p38γ KO inhibits human OS cell progression *in vitro*.** Expression of listed genes in the stable monoclonal OS1 cells, with the empty vector (“Cas9-C”) or the applied lenti-CRISPR/Cas9-p38γ-KO construct (with verified sgRNA, “sgRNA-1/-2”) was tested by qPCR and Western blotting assays (**A**, **B**). Cells were further cultured for applied time periods, cell proliferation (by measuring EdU ratio, **C**), migration (“Transwell” assay, **D**), invasion (“Matrigel Transwell” assay, **E**) and apoptosis (by measuring nuclear TUNEL ratio, **F**) were tested, and results quantified. Expression of listed proteins was quantified and normalized to the loading control (**B**). Data presented as mean ± standard deviation (SD, n=5). *** *p*< 0.001 vs. “Cas9-C” control cells. Experiments in this figure were repeated five times. Bar=100 μm (**C**–**F**).

To determine the effect of completely depleting p38γ, the CRISPR/Cas9 strategy was utilized to knockout (KO) p38γ. Two lenti-CRISPR/Cas9-GFPp38γ KO constructs(“sgRNA1”and “sgRNA2”) were individually transduced into the OS1 primary human OS cells. Stable p38γ KO OS1 cells were established, where *p38γ* mRNA (Figure 4A) and protein (Figure 4B) expression was depleted (*P*<0.001 vs. cells with control construct, Cas9). The applied p38γ KO constructs failed to alter p38α expression (*P*>0.05 vs. Cas9 control cells, Figure 4A, 4B). Similar to shRNA results, CRISPR/Cas9-induced p38γ KO inhibited OS1 cell proliferation (Figure 3C, *P*<0.001 vs. Cas9 control cells), potently decreased *in vitro* cell migration and invasion (Figure 4D, 4E, *P*<0.001 vs. Cas9 control cells), and caused significant apoptosis activation (Figure 4F, *P*<0.001 vs. Cas9 control cells). Collectively, these results show that CRISPR/Cas9-induced p38γ KO inhibited OS1 cell proliferation, migration and invasion, while inducing apoptosis activation.

### Ectopic overexpression of p38γ promotes human OS cell progression *in vitro*

**Figure 5:**
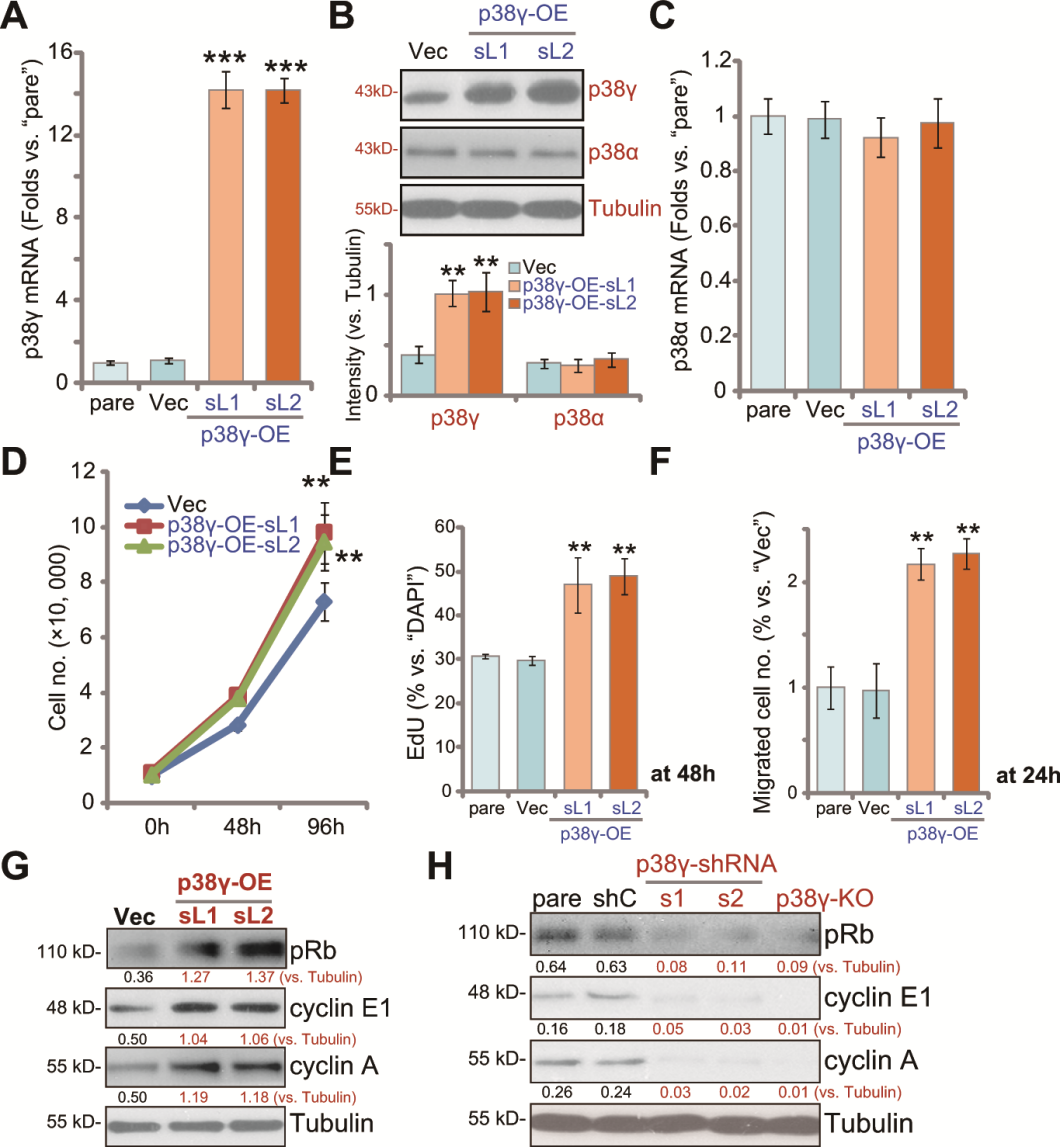
**Ectopic overexpression of p38γ promotes human OS cell progression *in vitro*.** Expression of listed genes in the stable OS1 cells, with the pLenti6-puro-GFP-p38γ expression vector (p38γ-OE-sL1 and p38γ-OE-sL2, two lines) or the empty vector (“Vec”), tested by Western blotting and qPCR assays (**A**–**C**); Cells were further cultured for applied time periods, cell growth (cell counting assay, **D**), proliferation (by measuring EdU ratio, **E**) and migration (“Transwell” assay, **F**) were tested; Rb phosphorylation and cyclin E1/A expression were tested by Western blotting (**G**). Rb phosphorylation and cyclin E1/A in the OS1 cells with scramble control shRNA (“shC”) or the applied p38γ shRNA (p38γ-shRNA-s1/s2), as well as in the p38γ-KO OS1 cells (by sgRNA-1), were tested and results were shown (**H**). Expression of listed proteins was quantified and normalized to the loading control (**B**, **G**, **H**). Data presented as mean ± standard deviation (SD, n=5). ** *p*< 0.01 vs. “Vec” cells.*** *p*< 0.001 vs. “Vec” cells. Experiments in this figure were repeated five times.

Based on the above results we hypothesized that ectopic p38γ overexpression would promote OS cell progression *in vitro*. A pLenti6-puro-GFP-p38γ expression vector (from Dr. Zheng at Nantong University [10]) was used to establish the two stable cell lines, p38γ-OE-sL1 and p38γ-OE-sL2. qPCR results, in Figure 5A, confirmed that *p38γ* mRNA levels were increased over 12 folds in the p38γ-OE cells (*P*<0.001 vs. Vector control cells/“Vec”), resulting in significantly increased levels of p38γ protein expression(Figure 5B, *P*<0.01 vs. “Vec” cells). In contrast, *p38α mRNA* (Figure 5C) and protein (Figure 5B) levels were unchanged in p38γ-OE OS1 cells (*P*>0.05 vs. “Vec” cells). Results show that p38γ overexpression promoted OS1 cell growth (Figure 5D), augmented cell proliferation (nuclear EdU incorporation, Figure 5E, *P*<0.01 vs. “Vec” cells) and migration (Figure 5F, *P*<0.01 vs. “Vec” cells), further supporting a key function of p38γ in OS cell progression.

p38γ has previously been reported to phosphorylate and inhibit Rb to promote expression of cyclin E1 and cyclin A [9, 10, 12]. In line with these findings, we found that Rb phosphorylation and cyclin E1/A expression were elevated in p38γ-OEstable OS1 cells (Figure 5G). Contrarily, in OS1 cells with p38γ-shRNA-s1/s2 (see Figure 2) and in p38γ-KO OS1 cells (by sgRNA-1, see Figure 4), Rb phosphorylation and cyclin E1/A expression were largely inhibited (Figure 5H).

## DISCUSSION

OS, often detected in children and young adults, is a genetically complex disease [6, 13, 14]. Genomic instability is a major contributor to OS disease progression and is associated with a poor prognosis [6, 13, 14]. The underlying molecular mechanisms of OS are still poorly understood [6, 13, 14]. The function of p38γ, an alternative p38 MAPK, has been disregarded in the studies and remains largely unknown [15]. Recent studies have identified novel p38γ substrates and new biological functions of p38γ [15]. It is implied that p38γ should have a tissue-specific function in tumor progression [9, 10, 15]. Tomas-Loba et al., demonstrated that p38γ exhibits high sequence homology, inhibition sensitivity and substrate specificity with known CDK family proteins [9, 12]. Thus, p38γ can function as a CDK-like kinase and cooperate with other CDKs to promote cell cycle entry [9, 12].

In the present study we show that mRNA and protein expression of p38γ are significantly elevated in human OS tissues and primary OS cells, compared to its low expression in primary human osteoblasts and OB-6 osteoblastic cells. Supporting a key function for p38γ in OS malignant behaviors, we found that overexpression of p38γ promoted human OS cell growth, proliferation and migration. Conversely, p38γ knockdown in both U2OS and primary human OS cells potently suppressed cell growth, proliferation, migration and invasion.

Yang et al., has shown that p38γ silencing by targeted siRNA reduced caspase-3/9 level and induced apoptosis in human glioma cells [16]. Furthermore, p38γ deletion led to S phase cell cycle arrest and cell apoptosis [17]. Recently, Chen et al., show that p38γ silencing or KO induced apoptosis activation in renal cell carcinoma (RCC) cells [10]. Furthermore, significant apoptosis activation in colorectal cancer cells was detected with p38γ silencing or KO [11]. In line with these studies, we demonstrated that p38γ silencing or KO induced significant apoptosis in established and primary human OS cells.

We found that Rb phosphorylation and cyclin E1/A expression were robustly inhibited with p38γ shRNA or KO, but augmented with ectopic p38γ overexpression. These results suggest p38γ-induced OS cell progression could be due to Rb inactivation and cyclin E1/A expression. In cancer cells, p38γ overexpression is reported to act as a non-classical CDK, promoting cell cycle progression by phosphorylating and inhibiting the tumor suppressor protein Rb [9, 10]. This in turn leads to the expression of cyclin A and cyclin E1, which are essential for cell cycle progression, cancer growth, proliferation and migration [9–11]. Cyclin A, which was originally proposed to function at the G2-to-M cell cycle transition, may have multiple functions in all aspects of cell cycle progression in human OS cells [18]. Notably, Molendini et al., showed that cyclin A overexpression in OS is associated with cancer relapse [19]. Cyclin E1 overexpression also has important prognostic and therapeutic implications for OS [20, 21].

Currently, the most effective clinical treatment regimens for OS include the combination of methotrexate, doxorubicin, and cisplatin (MAP) [2, 6–8]. The introduction of targeted therapies has so far failed to significantly improve the survival of OS patients [2, 6–8]. Our results suggest that p38γ is a novel and promising therapeutic target for this devastating malignancy. The dysregulation of cell cycle will lead to aberrant growth of OS cells, which is a characteristic hallmark of OS [22]. The primary proteins involved in cell cycle control are CDKs [22], emerging as key therapeutic targets. Indeed, CDK inhibitors are being developed to target OS cells [22]. Since p38γ is a novel CDK-like kinase important for OS cell progression, p38γ inhibitors are anticipated to exhibit potential anti-OS cell activity.

## MATERIALS AND METHODS

### Chemicals and reagents

Cell Counting Kit-8 (CCK-8) was obtained from Dojindo Co. (Kumamoto, Japan). Puromycin and Matrigel were provided by Sigma-Aldrich Chemicals (St. Louis, MO). Cell culture reagents, including fetal bovine serum (FBS) and antibiotics, were obtained from Hyclone Co. (Logan, UT). Antibodies of cleaved-caspase-3, cleaved-poly (ADP-ribose) polymerase (PARP), total PARP and tubulin were provided by the Cell Signaling Technology (Beverly, MA). All other antibodies were provided by Abcam Co. (Cambridge, UK).

### Cell culture

U2OS cells were purchased from the Cell Bank of Shanghai Institute of Biological Science (Shanghai, China), maintained under RPMI-1640 medium with 12% FBS. Three independent patient-derived primary human OS cells [23], namely OS1, OS2 and OS3, were from Dr. Ji at Nanjing Medical University [23], and cells cultured under the described conditions [23, 24]. The primary OS cells at passage 3-10 were utilized.OB-6 human osteoblastic cells were provided again by Dr. Ji [25] at Nanjing Medical University, cultured as descried [26]. The primary *human osteoblasts* were differentiated and cultured as described previously [27, 28]. The protocols of the study were approved by IACUC and Ethics committee of Soochow University.

### Human OS tissues

Human OS tumor tissues and the matched surrounding normal bone tissues from a total of twelve (12) written-informed OS patients were provided by Dr. Liang at Zhejiang University [29]. Tissues were incubated with the described lysis buffer [29], stored in liquid nitrogen. The protocols of the study were approved by Ethics committee of Soochow University.

### p38γ silencing by shRNA

GV248 (hU6-MCS-Ubiquitin-EGFP-IRES-puromycin) constructs expressing three different p38γ shRNAs (with non-overlapping sequences, p38γ-shRNA-s0/s1/s2) were provided by Dr. Cao at Fudan University [11], those were individually transduced to U2OS cells or the primary human OS cells for 48h. The stable cells were established by adding puromycin (5.0 μg/mL) in the complete medium for another 48h. In the stable cells*p38*γ mRNA and protein levels were tested. The scramble control shRNA was transduced to the control cells.

### Forced p38γ overexpression

A pLenti6-puro-GFP-p38γ expression vector (“OE-p38γ”) was provided by Dr. Zheng at Nantong University [10], transduced to primary human OS cells. Following selection using puromycin-containing complete medium, two stable cell lines (p38γ-OE-sL1 and p38γ-OE-sL2) were established, with p38γ overexpression verified by qPCR and Western blotting assays. Control cells were transduced with the vector control (“Vec”).

### p38γ knockout (KO)

CRISPR/Cas9 PX458-GFP constructs with p38γsmall guide RNA (“sgRNA-1” or “sgRNA-2”) were provided by again by Dr. Cao [11]. Each was individually transfected to OS cells. FACS was then carried out to sort the GFP-positive cells, which were distributed into 24-well plates. Using by qPCR and Western blotting assays, p38γ KO was screened, with stable monoclonal p38γ-KO OS cells established.

### Western blotting

The detailed protocols for Western blotting were previously described [27, 30]. The same set of lysates (40 μg per treatment) were run in parallel (“sister”) gels to examine different proteins. The total gray value of each band was quantified by using an ImageJ software (NIH, Bethesda, MD).

### Quantitative real-time reverse transcriptase polymerase chain reaction (qPCR) assay

The detailed protocols of qPCR, using a SYBR green kit under the ABI-7900 system, were described previously [27, 30]. The ΔΔCt method was utilized to quantify expression of targeted mRNAs, using *GAPDH* as the internal control [31]. All the primers utilized in this study were provided by Dr. Cao [11].

### Cell viability

Human OS cells with the applied genetic modifications were seeded into 96-well tissue culture plates (5 × 10^3^ cells per well). Following incubation for 72h, the cell viability was estimated by recording CCK-8’s optical density (OD) at 550 nm using a microplate reader.

### EdU (5-ethynyl-20-deoxyuridine) staining

Human OS cells with the applied genetic modifications were seeded into six-well plates (at 1 × 10^5^ cells in each well) and cultured for 48h. An EdU Apollo-567 assay kit (RiboBio, Guangzhou, China) was utilized to quantify cell proliferation. Briefly, cell nuclei were co-stained with EdU and DAPI for 3h, visualized under a fluorescent microscope (Leica, DM 4000, Germany)

### *In vitro* cell migration and invasion assays

Human OS cells (2 × 10^4^ cells/well of each condition) with the applied genetic modifications were seeded on the upper surface of “Transwell” chambers (8-mm pore, BD Biosciences, San Jose, CA) [32] in serum free medium. FBS-containing complete medium was added to the lower surface of “Transwell” chambers. After incubation for 24h, the migrated cells on the lower surface were stained and counted manually. To test cell invasion, Matrigel was always added to the “Transwell” chambers [33, 34].

### Cell cycle assay

Cells with applied genetic modifications were cultured for 48h, fixed and stained with propidium iodide (PI, 5μg/mL) and RNase. A flow cytometer (BD Biosciences, Franklin Lakes, NJ) was utilized to examine DNA contents. Cell cycle distribution was recorded, and results were quantified.

### Caspase-3 activity assay

Human OS cells with the applied genetic treatments were cultured for 36h, and a caspase-3 activity kit (Beyotime, Nantong, China) utilized to test caspase-3 activity. Briefly, 30 μg cytosolic protein lysates from each condition were incubated with caspase-3 assay buffer [35] and an AFC-conjugated caspase-3 substrate. After incubation for 2h under the dark, the AFC fluorescence intensity was quantified.

### Cell apoptosis detection

Human OS cells with the applied genetic modifications were seeded into six-well plates (at 1 × 10^5^ cells in each well) and cultured for 48h. The detailed protocols for cell apoptosis assays, including nuclear TUNEL [terminal deoxynucleotidyl transferase (TdT)-mediated dUTP nick end labeling] staining and Annexin V fluorescent-activated cell sorting (FACS), were described in our previous studies [27, 30].

### Statistical analysis

Data were presented as the mean ± standard deviation (SD). The difference between multiple groups was analyzed by ANOVA with multiple comparisons through Bonferroni post-hoc test, using a SPSS 21.0 software (SPSS Co., Chicago, IL). A two-tailed unpaired T test (Excel 2017) was applied to test significance between two groups. Values of *P*< 0.05 were considered statistically significant.
